# Emerging roles of SIRT6 and its modulators in kidney diseases

**DOI:** 10.1038/s41420-026-03264-y

**Published:** 2026-07-17

**Authors:** Feihong Ren, Yudian Wang, Yufei Zhang, Liheng Liu, Qi Jin, Liping Yang, Yongli Zhan

**Affiliations:** 1 Guang’anmen Hospital, China Academy of Chinese Medical Sciences, Beijing, China; 2 College of Traditional Chinese Medicine, Hubei University of Chinese Medicine, Wuhan, Hubei China; 3 China-Japan Friendship Hospital (Institute of Clinical Medical Sciences), Chinese Academy of Medical Sciences & Peking Union Medical College, Beijing, China

**Keywords:** Kidney diseases, Mechanisms of disease, Predictive markers

## Abstract

The global incidence and prevalence of kidney diseases continue to rise, posing a serious public health challenge. SIRT6 is an NAD⁺-dependent histone deacetylase with broad essential regulatory roles across various pathophysiological processes, including DNA repair, chromatin accessibility, telomere stability, and glycolipid metabolism. As an epigenetic regulator specifically expressed in kidney tissues, SIRT6 serves as a central mediator of kidney homeostasis. Notably, accumulating evidence has implicated aberrant SIRT6 expression in the onset and development of various kidney diseases, such as acute kidney injury, diabetic kidney disease, hypertensive nephropathy, renal fibrosis, and renal cell carcinoma. In the present review, we provide an overview of the sirtuin family, systematically characterize the enzymatic activities and critical biological functions of SIRT6, and discuss its molecular mechanisms across various kidney diseases, focusing on its cell type-specific functions. We further summarize the latest research advances in SIRT6-targeted modulators for improving kidney diseases and analyze the challenges associated with their clinical application. Overall, we highlight SIRT6 as a highly promising novel target in the treatment and prevention of kidney diseases, with strong potential for clinical translation.

**Figure:**
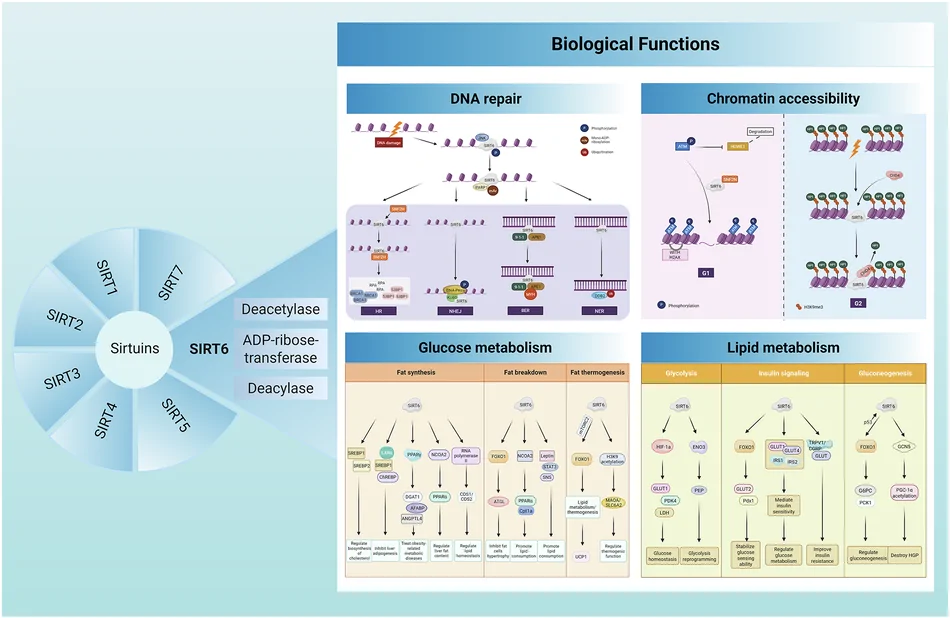

## Facts

- SIRT6 is a key member of the sirtuin family, a class of NAD⁺-dependent deacetylases, and possesses both ADP-ribose-transferase and deacylase activities.
- SIRT6 specifically modifies histone H3 to regulate various biological processes related to senescence, such as genomic integrity, DNA repair, energy metabolism, telomere maintenance, and gene expression.
- SIRT6 expression plays a key role in the kidneys and is closely related to the onset of various kidney diseases, including acute kidney injury, diabetic kidney disease, hypertensive nephropathy, and renal cell carcinoma.
- SIRT6 modulators serve as a promising strategy for treating kidney diseases.

## Introduction

Sirtuins, commonly referred to as longevity proteins, represent an evolutionarily conserved family of nicotinamide adenine dinucleotide (NAD^+^)-dependent deacetylases [1]. This family is the mammalian homolog of yeast Sir2 and includes seven members (SIRT1–SIRT7) [2]. Among these members, SIRT6 is an NAD^+^-dependent Class III histone deacetylase that serves as a core regulator of epigenetic homeostasis [3]. Structurally, it differs from other sirtuin family members by an N-terminal extension, a conserved catalytic core domain, and a C-terminal nuclear localization sequence. Moreover, SIRT6 engages NAD⁺ through a unique double-spherical structure [4]. This unique core structure of SIRT6 underlies its multifunctional enzymatic activity, encompassing deacetylation, mono-ADP ribosylation, and defatty acylation [5]. SIRT6 is specifically localized in the cell nucleus, where it participates in key processes, including DNA repair, telomere maintenance, chromatin accessibility, and energy metabolism. Through these functions, SIRT6 achieves dynamic integration of cellular metabolic signals and transcriptional programs [6–9].

The catalytic activity of SIRT6 is dependent on the cellular availability of NAD⁺, which renders its function closely linked to the state of cellular energy metabolism [10]. Under physiological or stress conditions, changes in the NAD⁺/NADH ratio regulate the catalytic efficiency of the SIRT6 enzyme, positioning this protein as a core sensor of metabolic status [11]. Notably, the kidney, as an important excretory and metabolic organ in the human body, carries substantial energy demands driven by its continuous processes of filtration, reabsorption, and endocrine regulation; these functions collectively contribute to maintaining homeostasis [12, 13]. Given the role of the SIRT6-mediated metabolic sensing pathway in maintaining renal homeostasis, SIRT6 is particularly crucial in renal physiology and pathology. Recently, an increasing body of evidence has demonstrated that SIRT6 dysregulation is implicated in various kidney diseases, such as acute kidney injury (AKI), diabetic kidney disease (DKD), hypertensive nephropathy (HN), renal fibrosis, and renal cell carcinoma (RCC) [14–16].

Therefore, by synthesizing the latest research advances, this review aimed to comprehensively elaborate on the enzymatic activity and core biological functions of SIRT6, focusing on its molecular mechanisms across different kidney diseases, including cell-specific mechanisms. Furthermore, this review highlights the application prospects and translational challenges of targeting SIRT6 in delaying the progression of kidney diseases.

## The sirtuin family

SIRT1–7 can precisely modulate specific lysine (K) residues on histones to mediate post-translational modifications, thus promoting chromatin silencing and transcriptional repression [17]. Deacetylation also targets non-histone proteins and subsequently regulates their functional activities [18]. Sirtuins couple their enzymatic activities to cellular energy metabolism by depending on NAD⁺ [19]. Specifically, NAD⁺ and its reduced form NADH govern the redox balance during cellular energy synthesis [19]. However, prolonged oxidative stress depletes NAD⁺ pools, diverts them toward the repair of DNA damage due to reactive oxygen species, and ultimately suppresses the activity of sirtuins [19]. Other enzymes, such as ADP-ribosyl cyclases, which hydrolyze NAD⁺ to nicotinamide and ADP-ribose, may also affect the bioavailability of NAD⁺ [20].

**Fig. 1:**
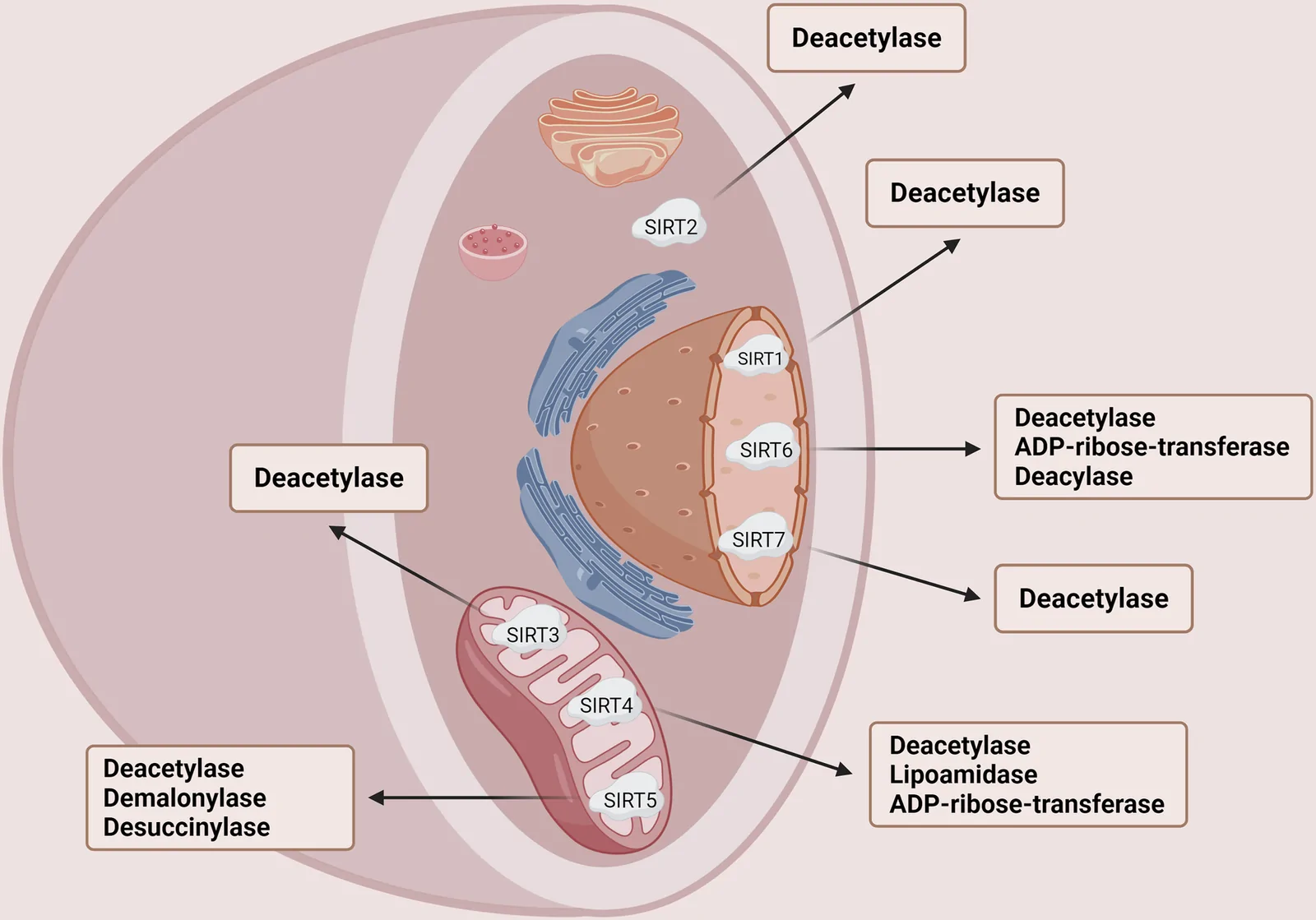
Localization and enzymatic activities of sirtuin family members in mammals. Mitochondrial sirtuins include SIRT3, SIRT4, and SIRT5. SIRT3 mainly exerts deacetylase activity; SIRT4 mainly functions as a deacetylase, lipoamidase, and ADP-ribose-transferase; and SIRT5 mainly functions as a deacetylase, demalonylase, and desuccinylase. Nuclear sirtuins include SIRT1, SIRT6, and SIRT7. SIRT1 possesses deacetylase activity; SIRT6 mainly acts as a deacetylase, ADP-ribose-transferase, and deacylase; and SIRT7 mainly exerts deacetylase activity. SIRT2 is located in the cytoplasm, where it acts as a deacetylase.

Despite sharing the identical cofactor to enhance deacetylase activity, sirtuins exhibit different functions, primarily owing to differences in subcellular localization and the wide range of their target protein repertoires (Fig. 1). In the following section, we summarize nuclear, cytoplasmic, and mitochondrial sirtuins.

### Nuclear sirtuins

Among the sirtuin family members, SIRT1 is the most investigated and is involved in regulating nucleosome histone acetylation [21]. Following oxidative stress and DNA damage, SIRT1-dependent p53 deacetylation decreased cell apoptosis and senescence [22]. Moreover, SIRT1 can modulate tumor necrosis factor-alpha (TNF-α)-induced inflammation, the activity of hypoxia-inducible factor-2α (HIF2α), and the expression of genes regulated by peroxisome proliferator-activated receptor γ (PPARγ) [23–25]. SIRT6, which is also present in the cell nucleus, deacetylates histone H3 at K9 and K56 to maintain genomic stability and proper telomere functioning [26]. SIRT6 promotes DNA repair, and the SIRT6-knockout cell model exhibits hypersensitivity to DNA damage and genomic destabilization [27]. SIRT7 is the only sirtuin predominantly localized to the nucleolus and is necessary for ribosomal DNA transcription [28]. A recent study indicated that SIRT7 directly interacts with the chromatin remodeling complex to control RNA polymerase I-mediated transcription [29]. SIRT7 can also inhibit energy expenditure and thermogenesis by regulating brown adipose tissue and uncoupling protein 1 (UCP1) expression [30].

### Cytoplasmic sirtuins

Although SIRT2 is located in the cytoplasm, it can translocate to the nucleus during microtubule-related mitosis [31]. SIRT2 binds to Forkhead box O3a (FOXO3a) to promote its nuclear transport, induces the expression of caspases-3 and -8, and initiates the apoptotic process [32]. Moreover, SIRT2 contributes to the nuclear stabilization of p53 and enhances its transcription by modulating its acetylation status at the lysine 382, thereby regulating genes involved in the DNA damage response [33].

### Mitochondrial sirtuins

SIRT3 is localized to the mitochondria and serves as a key regulator of the whole organelle acetylome [34]. SIRT3 mediates FOXO3a deacetylation, enhances its nuclear localization, and promotes the expression of FOXO3a-dependent peroxidase, thereby protecting cells from damage due to oxidative stress [35]. Beyond its role in regulating mitochondrial antioxidant pathways, SIRT3 also governs energy production in various ways. During membrane depolarization, SIRT3 dissociates from complex V to promote the deacetylation of specific protein clusters and significantly optimize energy production efficiency [36]. SIRT4 mediates post-translational modifications of proteins through multiple mechanisms, including deacetylation and ADP-ribosyltransferase activity [37]. SIRT4 promotes ADP ribosylation, suppresses glutamate dehydrogenase activity, and inhibits the conversion of glutamate to α-ketoglutarate in the tricarboxylic acid cycle [38]. SIRT5 primarily regulates lysine acylation and functions as a desuccinylase and a deglutarylate [39]. Upon DNA damage, SIRT5 mediates p53 desuccinylation at lysine 120, thereby suppressing its transcription and function [40]. Importantly, two key molecules regulate SIRT5 expression: activation of AMP-activated protein kinase (AMPK) downregulates SIRT5 expression, whereas overexpression of peroxisome proliferator-activated receptor γ coactivator 1-α (PGC-1α) upregulates SIRT5 expression and activity [41].

## Molecular structure and enzymatic activity Of Sirt6

### Molecular structure of SIRT6

Mammalian SIRT6 is a medium-sized protein consisting of 355 amino acids, with a predicted molecular weight of 39.1 kDa and an isoelectric point of 9.12 [42]. The SIRT6 gene comprises eight exons, of which exon 8 is the longest (838 bases), and exon 4 is the shortest (60 bases) [42]. Structurally, SIRT6 contains an N-terminal extension (NTE; residues 1–42), a conserved catalytic core domain (residues 43–276), and a C-terminal extension (CTE; residues 277–355) [43]. While the CTE is essential for proper nuclear localization of SIRT6, it is not required for catalytic activity [43]. The terminal seven-amino acid sequence of the CTE, 345—^345^PKRVKAK^351^, corresponds to a classical nuclear localization signal, indicating its role in nuclear localization. In contrast, the NTE is pivotal for the intrinsic catalytic activity of SIRT6 and plays a key role in nucleosome binding, thereby facilitating chromatin association [43]. Therefore, mutants with N-terminal deletions exhibit a drastically reduced ability to regulate histone H3 lysine 9 (H3K9) and H3K56 acetylation levels in cells. Moreover, the enzymatic activity and chromatin association of SIRT6 are based on the enzymatic core domain, so that is why these roles are abrogated once the H133Y mutation occurs there [43].

Similar to other three-dimensional structures in the sirtuin family, SIRT6 possesses two main globular domains: a large Rossmann fold that mediates NAD^+^ binding and a relatively small zinc-binding motif [44, 45]. The Rossmann fold consists of a six parallel β-sheet (β1, β2, β3, β7, β8, and β9) flanked by four α- helices (a1, a4, a5, and a8) on one side and two α- helices (a6 and a7) on the other. The small zinc-binding domain comprises three antiparallel β-sheets (β4, β5 and β6) stabilized by the loops of two α-helices (α3 and α6) [46]. Even in the absence of substrates, SIRT6 with a single helix adopts conformations that confer high affinity for NAD^+^ binding [44, 46]. Notably, the long-chain myristoyl moiety of the acylated peptide myristoyl H3K9 is accommodated within a hydrophobic channel formed by hydrophobic residues across multiple flexible loops, including the N-terminal region [47]. In vitro, SIRT6 exhibits a relatively weak deacetylase activity and preferentially hydrolyzes long-chain acylated peptides; this property may be attributed to the unique configuration between the Rossmann fold and the zinc-binding domain [48]. In addition, free fatty acids enhance the catalytic efficiency of SIRT6 stimulated by approximately 35-fold, likely owing to the competitive binding of free fatty acids to the hydrophobic channels occupied by the acyl moiety of the acylated substrate [48].

### Enzymatic activity of SIRT6

#### Deacetylation

Histone deacetylation is one of the most investigated post-translational modifications. It is intrinsically related to cellular metabolism, transcription initiation, cell cycle control, DNA damage repair, telomeric maintenance, and the modulation of genomic architecture [49]. As mentioned above, SIRT6 is an NAD^+^-dependent histone deacetylase that targets lysine residues in nuclear histones (Table 1). [50]. Michishita et al. were the first to demonstrate that human SIRT6 is an H3K9 deacetylase that can regulate telomeric chromatin [51]. Notably, SIRT6 exhibits the most stable deacetylation activity toward peptides containing acetylated H3K9. The loss of SIRT6 results in telomere dysfunction, characterized by end-to-end chromosomal fusions and premature cellular senescence, highlighting its critical role in establishing and maintaining specific chromatin states at mammalian telomeres [51]. Tiara et al. further confirmed that SIRT6 interacts with the RELA subunit of nuclear factor kappa-B (NF-κB), promoting H3K9 deacetylation at the promoters of NF-κB target genes and attenuating NF-κB signaling. Through this mechanism, SIRT6 regulates gene expression, apoptosis, and cellular senescence [52].

**Table 1:** The substrates and functions of SIRT6.

Enzymatic action	Substrates		Site	Physiological function	References
**Deacetylation**	Histones	H3K9	K9	Regulate telomere chromatin	[51]
		H3K9	K9	Reduce NF-κB signaling	[52]
		H3K9	K9	Regulate glycolysis and HIF1α	[53]
		H3K9	K9	Inhibit Notch and protect podocytes	[54]
		H3K9	K9	Inhibit the transcription of Nkx3.2 and regulate gata5	[55]
		H3K9	K9	Antagonize Ero1α mediated by HIF1α/ p300	[56]
		H3K56	K56	Regulate the elongation of transcription	[58]
		H3K56	K56	Regulate the binding with IL-1	[59]
		H3K56	K56	Prevent DNA damage	[8]
		H3K18	K18	Prevent mitotic errors and cellular senescence	[60]
	Non-histones	PKM2	K433	Inhibit PKM2 nucleoprotein kinase	[61]
		Ku70	K542	Block the mitochondrial translocation of Bax	[62]
		STAT5	K163	Suppress the IL-15/ JAK3/STAT5 signal	[63]
		SMAD2	K54	Participate in the activation of HSCs	[64]
		SMAD3	K333/378	Prevent liver fibrosis	[65]
		GCN5	K549	Inhibit hepatic glucose production	[66]
		NAMPT	K53/369	Regulate NAMPT and eNAMPT	[67]
		SOD2	K68/122 K63/209	Regulate depressive responses	[68]
		RORγt	K192	Regulate IL-17A-induced inflammatory chemokines and interstitial reprogramming	[69]
		hnRNPA2B1	K113/173	Lead to local transcription inhibition	[70]
		NOS3	K494/497/504	Regulate endothelium-dependent relaxation of the arterial wall	[71]
**Mono-ADP-ribosylation**	Non-histones	SIRT6	/	Regulate SIRT6 activity	[73]
		PARP1	K521	Promote the repair of DSBs	[7]
		p53/p73	/	Regulate the apoptosis of cancer cells	[74]
		KAP1	/	Participate in the senescence process	[75]
		BAF170	K312	Mediate the formation of active chromatin loops and regulate oxidative stress	[76]
		JHDM1A/KDM2A	/	Regulate transient inhibition of transcription initiation and repair of DNA DSBs	[77]
		LMNA	/	Improve the longevity genome	[78]
		YY1	K409	Reduce degeneration of the neuromuscular junction	[79]
**Defatty-acylation**	Histones	H3K9, H3K18, H3K27	K9/18/27	/	[84]
	Non-histones	TNF-α	K19/20	Accelerate the secretion of TNF-α	[47]
		R-Ras2	/	Inhibit the membrane localization of R-Ras2 and the proliferation of downstream cells	[83]

SIRT6 functions as a key inhibitor of the transcription factor HIF1α and the expression of multiple glycolytic genes [53]. SIRT6-deficient cells only exhibit upregulated HIF1α activity and elevated glucose uptake due to reduced mitochondrial respiration and increased glycolysis. These data indicate that SIRT6 is the main regulator of glucose homeostasis [53]. Moreover, Liu et al. demonstrated that SIRT6 inhibits the transcription of Notch1 and Notch4 signaling by deacetylating H3K9 at their target loci, thereby enhancing autophagic flux and protecting podocytes from apoptosis and inflammation [54]. Guo et al. demonstrated that SIRT6 maintains endothelial function by deacetylating H3K9, suppressing the transcription of NK3 homobox 2, and modulating the GATA-binding protein 5-mediated signaling pathway [55]. In addition, by deacetylating H3K9, SIRT6 prevents the recruitment and accumulation of HIF1α and p300 at the promoter or endoplasmic reticulum oxidase 1 alpha (ERO1Α), thereby antagonizing HIF1α/P300-mediated ERO1Α transcription and preserving the barrier function of cardiac microvascular endothelial cells [56].

H3K56 is another chromatin-associated substrate of SIRT6. Compared to SIRT1, SIRT6 is more effective in deacetylating H3K56. Physiologically, H3K56 deacetylation is crucial, as it is implicated in the regulation of genomic stability, DNA replication and repair, and telomeric chromatin stability [57]. In SIRT6-knockout cells and in vivo knockout models, the levels of H3K56Ac are significantly elevated, and H3K56 hyperacetylation in SIRT6-deficient cells can be reversed by reconstitution with wild-type (WT) SIRT6. Moreover, SIRT6 regulates polymerase II (Pol II) activity by inhibiting the release of negative elongation factor and stabilizing promoter-proximal Pol II pausing through the deacetylation of H3K9 and H3K56, thereby restraining transcription elongation [58]. Furthermore, chromatin immunoprecipitation analyses revealed that SIRT6 binds to the interleukin (IL)-1 promoter and partially suppresses its expression by reducing H3K9 and H3K56 acetylation levels [59]. SIRT6 also recruits the chromatin remodeler SNF2H to sites of double-strand breaks (DSBs), where it promotes H3K56 deacetylation and coordinates the DNA damage response to preserve genomic integrity [8].

In addition to the well-established deacetylation of H3K9 and H3K56, human SIRT6 also promotes the deacetylation of H3K18 at pericentric heterochromatin. SIRT6-mediated H3K18 deacetylation plays a key role in preventing cellular senescence, mitosis defects, and genomic instability [60]. The transcriptional corepressor KRAB-interacting protein 1 (KAP1) serves as an intermediate in H3K18 deacetylation. KAP1 specifically binds unacetylated histone H3, whereas H3K18 acetylation disrupts this interaction. In SIRT6-knockout cells, H3K18 hyperacetylation stimulates KAP1 release and transcriptional inhibition.

Given that H3K9, H3K56, and H3K18, as SIRT6 substrates, are subject to SIRT6-mediated deacetylation, some non-histone proteins may similarly serve as SIRT6 substrates and undergo deacetylation to regulate intracellular signaling pathways [Table 1]. For example, SIRT6 can deacetylate nuclear pyruvate kinase M2 at K433 and inhibit its nuclear protein kinase and transcriptional coactivator functions. This inhibition results in decreased cell proliferation, migration, and invasiveness, highlighting the key role of SIRT6 as a tumor suppressor [61]. Although SIRT6 does not regulate the expression of Ku70, it interacts with Ku70 to deacetylate lysine K542. This modification contributes to blocking the mitochondrial translocation of BCl2-associated X protein (Bax), thereby reducing apoptosis in hepatocellular carcinoma (HCC) cells [62]. SIRT6 also directly interacts with STAT5, deacetylating the conserved lysine 163 to limit the IL-15/JAK3-mediated nuclear translocation of STAT5 and attenuating chondrocyte senescence [63]. Zhang et al. reported that SIRT6 deacetylates conserved K54 on mothers against decapentaplegic homolog (Smad) 2, downregulates the transcription of transforming growth factor-β (TGF-β)/Smad2 signaling, and modulates the activation of hepatic stellate cells. These findings suggest its potential as a therapeutic target in liver fibrosis [64]. SIRT6 is also involved in the deacetylation of the key lysine residues K333 and K378 of SMAD3; it inhibits the TGF-β-induced transcriptional activity of SMAD3 in hepatic stellate cells [65]. Moreover, SIRT6 directly interacts with and modifies general control nonrepressed protein 5 (GCN5), targeting residue K549. This targeting promotes PGC-1α acetylation to inhibit hepatic glucose production [66]. SIRT6 may also enhance intracellular NAD phosphate levels by modulating the enzymatic activity of nicotinamide phosphoribosyltransferase (NAMPT) and the secretion of extracellular NAMPT through differential acetylation at K53 and K369, respectively, thereby conferring protection against oxidative stress [67]. SIRT6 binds directly to superoxide dismutase 2 (SOD2) and peroxidoredoin 6 (Prdx6) and deacetylates them at Lys68/122 and Lys63/209 respectively, which are subsequently demonstrated to be acetylated by p300/ CBP-related factor (PCAF) and are essential for the antidepression response [68]. Furthermore, the specific deletion of SIRT6 in airway epithelial cells decreases the interaction with RORγt, inhibits its deacetylation at K192 mediated by the PPXY motifs, and prevents its recruitment into the IL-17A promoter. These changes regulate IL-17A-induced inflammatory chemokine signaling and mesenchymal reprogramming, thus preventing airway inflammation and remodeling mediated by allergens [69]. Moreover, SIRT6 promotes the deacetylation of hnRNPA2B1 at K113/173 residues, enabling it to rapidly disassociate from DSBs. This dissociation, in turn, disrupts the integrity of the RNA polymerase II complex on active chromatin, resulting in local transcription repression [70]. Finally, SIRT6 promotes the expression of NOS3 by upregulating Kruppel-like factor 2 expression and deacetylates NOS3 at K494, K497, and K504 of the calmodulin-binding domain, which enhances NOS3 activity and regulates endothelium-dependent relaxation of the arterial wall [71].

#### Mono-ADP-ribosylation

Although SIRT6 is the most extensively studied histone deacetylase, the first description of its enzymatic activity suggested that SIRT6 functions as a mono-ADP-ribosyltransferase (Table 1). [72]. Early in vitro experiments revealed that purified recombinant mouse SIRT6 (mSIRT6) significantly catalyzes the transfer of radiolabel from [32 P]NAD to mSIRT6; this process demonstrates the ADP-ribosyltransferase activity and requires two highly conserved residues in the protein catalytic core [73]. In WT mSIRT6, two key mutations completely abolished the approximately 15% high labeling efficiency, indicating that radiolabel transfer excluded covalent interaction between [32 P] NAD+ and mSIRT6 and was dependent on enzymatic activity. Importantly, SIRT6 transfers the radiolabel via an intramolecular mechanism, suggesting that SIRT6 may utilize ADP ribosylation as a means of auto-regulating its activity. However, the physiological value of auto-ADP ribosylation requires further exploration. Mao et al. confirmed that SIRT6-mediated ADP ribosylation is also involved in the process of DNA DSB repair [7]. Under oxidative stress in mammalian cells, SIRT6 is recruited to DNA DSB sites, where it binds poly ADP-ribose polymerase 1 (PARP1) and catalyzes mono-ADP ribosylation at K521, thereby triggering PARP1 ADP-ribosylase activity and enhancing DSB repair efficiency. Another study demonstrated that SIRT6 activation may be closely related to cancer cell apoptosis. Specifically, SIRT6 promotes apoptosis across various cancer cell lines by inducing the p53 and p73 apoptotic signaling cascades; notably, this pro-apoptotic function is mediated specifically by the mono-ADP-ribosyltransferase activity of SIRT6 rather than its deacetylase activity [74]. In cancer cells, blocking ATM signals significantly downregulates SIRT6-mediated apoptosis. In addition, Michael et al. reported that SIRT6 physically associates with the 5′-untranslated region of long interspersed element 1 (L1) loci and contributes to their transcriptional repression. It promotes the interaction between the nuclear corepressor protein KAP1 and the heterochromatin factor HP1α through its mono-ADP-ribosyltransferase activity, facilitating heterochromatin formation at L1 loci. This epigenetic silencing suppresses L1 retrotransposition, highlighting the role of SIRT7 in aging [75]. Moreover, SIRT6 can mono-ADP-ribosylate the BAF chromatin remodeling complex subunit (BAF170) at K312. This post-translational modification promotes the recruitment of BAF170 to the enhancer region of the heme oxygenase-1(HO-1) locus, mediating the formation of an active chromatin loop at this site. This organization is directly associated with the activation of NF-E2-related factor 2 (Nrf2)-dependent antioxidant response genes under oxidative stress [76]. Sarallah et al. discovered that SIRT6 mediates the mono-ADP-ribosylation of the histone demethylase JHDM1A/KDM2A, promoting the rapid disassociation of KDM2A from chromatin. This relocalization leads to elevated H3K36me2 levels, subsequent H3K9 tri-methylation, and ultimately transient suppression of transcription initiation and DNA DSB repair [77]. In a survey involving 450 centenarians and 550 individuals without a family history of exceptional longevity, targeted sequencing of the SIRT6 locus revealed significant enrichment of SIRT6 alleles with two linkage substitutions (N308K/A313S) among centenarians [78]. Notably, these SIRT6 variants exhibited enhanced mono-ADP-ribosyltransferase activity but reduced deacetylase activity. Functionally, they also promoted the ribosylation of Lamin A, thus improving the longevity-associated cellular phenotypes. A recent study demonstrated that SIRT6 can mono-ADP-ribosylate the dystrophin repressor YY1, resulting in its dissociation from the dystrophin promoter. Following this displacement, YY1 interacts with the E3 ubiquitin ligase SMURF2, resulting in its ubiquitin-mediated degradation. This pathway effectively attenuates the deleterious consequences of the degeneration at the neuromuscular junction [79]. Moreover, loss of protein kinase C delta results in the upregulated expression of the downstream mediator SIRT6, stimulates chromatin-associated H3K36me2, and decreases KDM2A ribosylation, thus suggesting a new pathway for DNA repair [80]. Fucose-FV also facilitates DNA repair and inhibits senescence by promoting the deacetylation and mono-ADP-ribosylation activities of SIRT6 [81].

#### Defatty-acylation

Although SIRT6 exhibits deacetylation activity, it is approximately 1000-fold weaker than that of other members of the sirtuin family [46]. The weak in vitro deacetylase activity of SIRT6 suggests that it may possess other enzymatic functions. For example, in addition to its well-known NAD-dependent deacetylation and mono-ADP-ribosylation activities, SIRT6 also possesses defatty-acylase activity (Table 1). [48]. The SIRT6 mutant (G60A) exhibits effective defatty-acylase activity, whereas its deacetylase activity is markedly reduced in vitro and is not detectable in cells [82]. This defatty-acylase activity of the G60A mutant promotes the secretion of various proteins [82]. Despite the potential role of SIRT6 in promoting protein secretion, research on its defatty acylase activity remains limited compared to that on its histone deacetylase activity. For example, TNF-α is among the earliest identified target proteins for SIRT6-dependent deacylation to be extensively studied. Jiang et al. revealed that the crystal structure of human SIRT6 exhibits a large hydrophobic pocket that can accommodate long-chain fatty acyl groups, effectively eradicating long-chain fatty acyl groups from lysine residues [47]. Structurally, SIRT6 promotes the secretion of the pro-inflammatory cytokine TNF-α by removing the fatty acyl modifications on residues K19 and K20 of TNF-α. The mechanism by which SIRT6 regulates TNF-α secretion suggests that SIRT6 may localize to secretory organelles, such as the endoplasmic reticulum. Similar SIRT6-dependent regulatory mechanisms may also apply to other secreted proteins, including insulin-like growth factor 1. In addition, SIRT6 regulates the lysine fatty acylation of the Ras family member R-Ras2 [83], further highlighting its role in modulating protein function through diacylation. Specifically, in mouse embryonic fibroblasts (MEFs), SIRT6 knockout leads to an increase in the lysine fatty acylation of R-Ras2 and facilitates the plasma membrane localization of R-Ras2 and its interaction with phosphatidylinositol 3-kinase (PI3K), thus activating the PI3K/Akt signaling pathway. This activation, in turn, promotes cell proliferation. In contrast, SIRT6 disrupts this plasma membrane localization ability of R-Ras2 by removing its fatty acyl modifications, inhibiting downstream proliferative signaling. By combining recombinant histone H3 synthesis with chemical biology approaches, Wang et al. demonstrated that SIRT6 can effectively and rapidly remove the fatty acyl groups from H3K9, H3K18, and H3K27, whereas its defatty-acylation activity toward H3K14, H3K36, H3K5,6 and H3K79 is comparatively weak [84]. In addition, SIRT6-mediated defatty acylation regulates the secretion of 497 proteins, including COL6A1, COL5A1, RPL17, and RPS7 [82]. Specifically, the defatty-acylation activity of SIRT6 inhibits the exosomal sorting of the ribosomal proteins RPL17 and RPS7, thereby preventing their secretion. More recently, 2-methyl-N-(4-phenoxy-phenyl)pyrazolo [1,5-a]quinazoline-5-amine (21q), a new potent SIRT6 activator, was demonstrated to significantly enhance the defatty-acylation activity of SIRT6, improve its thermal stability, and suppress the PI3K/Akt pathway, thus inhibiting the proliferation of MEFs [85]. Although research on SIRT6-mediated defatty acylation has expanded our understanding of its role in protein modification and other regulatory functions, the comprehensive molecular mechanism underlying this activity remains to be fully characterized.

## Biological functions Of Sirt6

### DNA repair

**Fig. 2:**
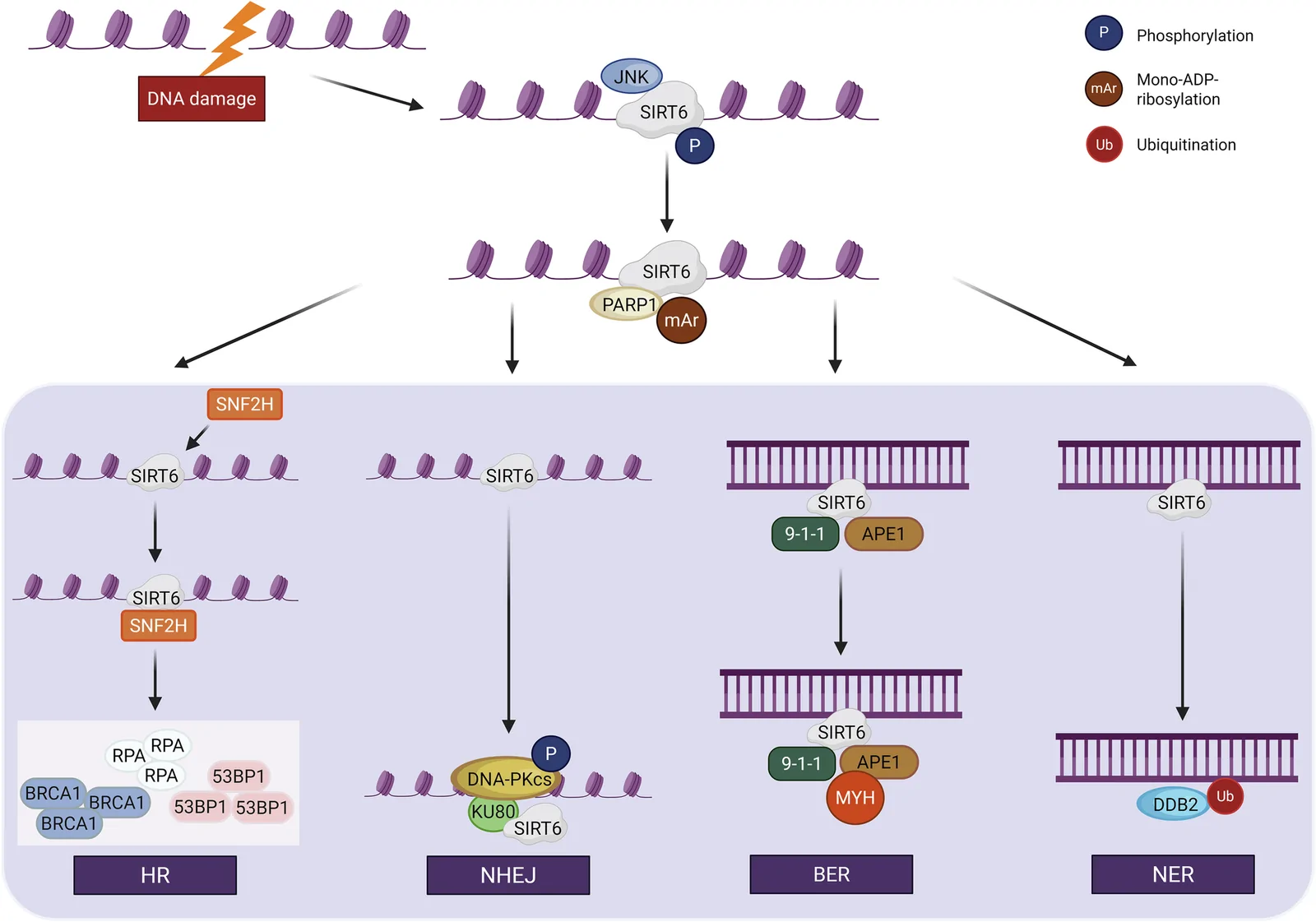
DNA repair function of SIRT6. SIRT6 is involved in the DNA repair process through homologous recombination (HR), non-homologous end joining (NHEJ), base excision repair (BER), and nucleotide excision repair (NER). JNK c-Jun N-terminal kinase, PARP1 poly(ADP-ribose) polymerase 1, BRCA1 breast cancer 1, RPA replication protein A, DNA-PKcs DNA-PK catalytic subunit, 9-1-1 Rad9-Rad1-Hus1, APE1 AP endonuclease 1, MYH MutY homolog, DDB2 DNA-binding protein 2.

Numerous members of the sirtuin family are intrinsically involved in DNA repair processes [27]. For example, SIRT6 interacts with nuclear chromatin in response to DNA damage in cells [86], and SIRT6-deficient cells exhibit genomic instability and hypersensitivity to DNA damage, indicating that SIRT6 plays a key role in DNA repair [Fig. 2] [87]. To address the various forms of DNA damage, such as DSBs, single-strand breaks, nicks, and gaps, cells have evolved multiple repair mechanisms, each tailored to a specific type of damage. For example, DSBs, a deleterious form of DNA damage, are primarily repaired through two major mechanisms: homologous recombination (HR) and non-homologous end joining (NHEJ). In contrast, DNA bases that have undergone minor chemical modifications are repaired through base excision repair (BER), which recognizes and removes aberrant bases before they can compromise genomic integrity.

Under oxidative stress conditions, activated protein kinase c-Jun N-terminal kinase phosphorylates SIRT6 at the serine(S)10 site, and this post-translational modification promotes SIRT6 recruitment to DNA cleavage sites [88]. PARP1 specifically interacts with SIRT6 to regulate DNA cleavage sites and facilitate DSB repair. Notably, SIRT6 overexpression significantly enhances DSB repair efficiency, as evidenced by a 3.4-fold increase in HR repair efficiency and a 3.3-fold increase in NHEJ efficiency [88]. During replicative cell senescence, HR repair efficiency progressively declines, and the predominant repair strategy shifts toward the NHEJ pathway.

SIRT6 has been established as a key factor in the early recruitment response to DSB sites [8]. Upon DSB induction, SIRT6 deacetylates histone H3K56 and recruits the chromatin remodeler SNF2H to the damage site [8]. Together, SIRT6 and SNF2H drive chromatin remodeling and the downstream recruitment of repair factors, such as 53BP1, RPA, and breast cancer 1 [8]. Notably, SIRT6 and SNF2H cooperate with ATM to inhibit the ubiquitination of H2AX and its subsequent proteasomal degradation, thus promoting its incorporation into chromatin specifically at the DSB sites [89].

In response to DNA damage, SIRT6 forms complexes with the repair factor DNA-dependent protein kinase (DNA-PK) and promotes the recruitment of its catalytic subunit (DNA-PKcs) to chromatin, thereby promoting effective DSB repair [27]. In induced pluripotent stem cells (iPSCs), SIRT6 can directly bind to Ku80, enhance the interaction between Ku80 and DNA-PKcs, and promote the phosphorylation of DNA-PKcs, highlighting its role in NHEJ repair [90].

SIRT6 also plays a regulatory role in the BER pathway, and its mechanism involves modulating core BER factors and chromatin accessibility. SIRT6 promotes BER in a PARP1-dependent manner, and its overexpression prevents the age-associated decline in BER efficiency in aged fibroblasts [91]. Under conditions of oxidative DNA damage, SIRT6 forms a complex with MYH glycosylase, AP endonuclease 1 (APE1), and Rad9-Rad1-Hus1 (9-1-1) that helps maintain genomic and telomeric integrity in mammalian cells [92]. Specifically, MYH and APE1, as BER enzymes, can help prevent mutations induced by oxidative DNA damage, and 9-1-1 promotes cell cycle checkpoint signaling and DNA repair [92]. Notably, SIRT6 mediates the deacetylation of lysine residues in DNA-binding protein 2, promoting its ubiquitination and dissociation from chromatin, and thus inducing the nucleotide excision repair process [93].

### Chromatin accessibility

**Fig. 3:**
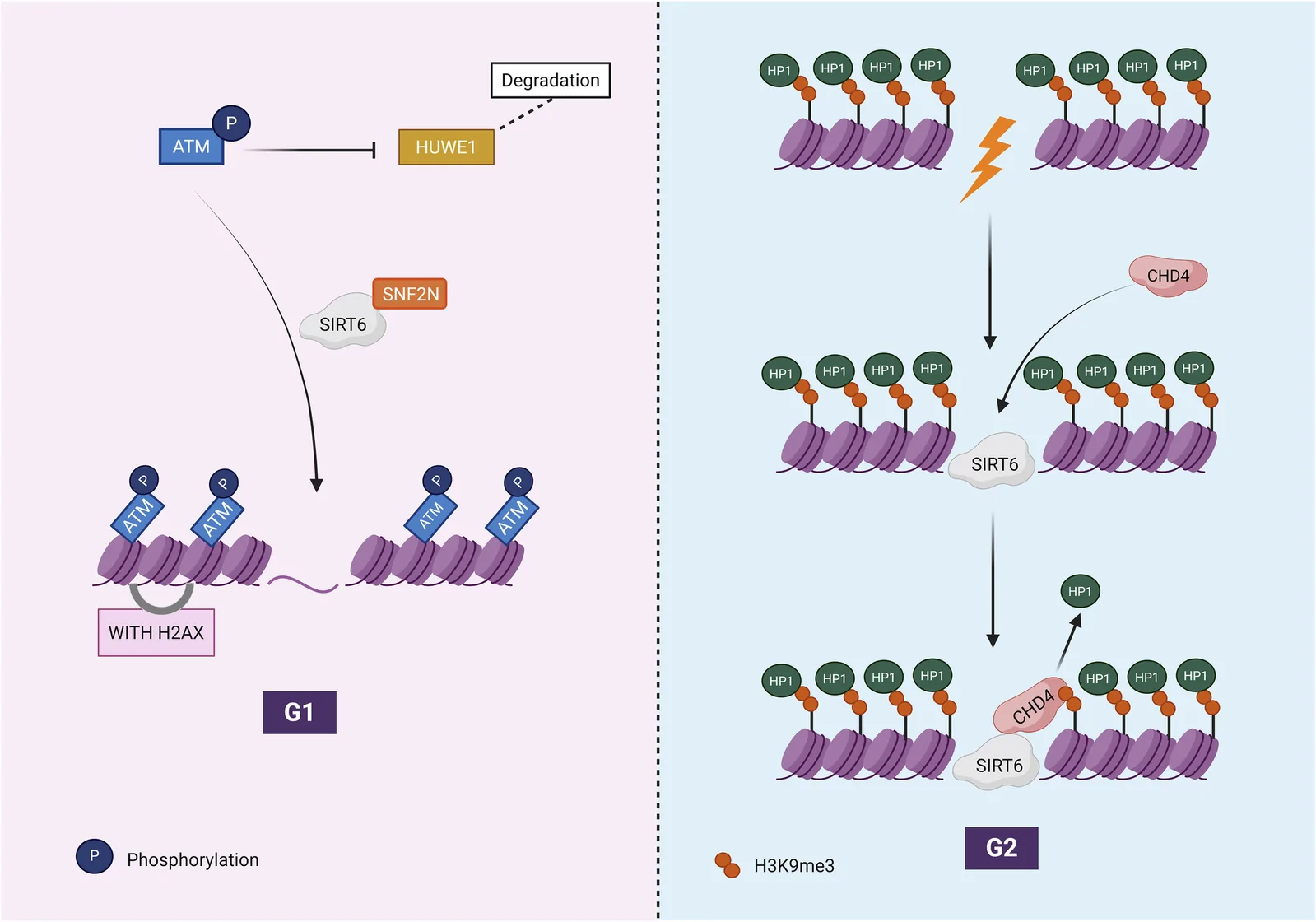
SIRT6 plays a role in regulating chromatin accessibility. HP1 heterochromatin protein 1.

Chromatin is composed of DNA wrapped around histone proteins to form nucleosome structures. When nucleosomes are tightly packed, DNA remains condensed, and chromatin adopts a “closed” conformation, restricting the access of DNA repair factors to DNA damage sites [94]. Therefore, chromatin accessibility, as a dynamically regulated epigenetic feature, plays a core role in the precise regulation of the DNA damage response (DDR) [95]. Chromatin remodeling factors participate in the DNA repair process by modifying chromatin structure via histone acetylation or deacetylation modifications [96, 97]. As mentioned previously, SIRT6 can reshape chromatin and prevent genomic instability by inducing H3K56 deacetylation and recruiting SNF2H into DSB sites. The ATM–SIRT6/SNF2H complex regulates transient H2AX stability by blocking the E3 ubiquitin ligase HUWE1 and enters chromatin only upon DNA damage induction [89]. Once SIRT6 localizes to the DNA damage site, CHD4 competitively displaces heterochromatin protein 1 from H3K9me3, implicating this pathway in HR-mediated DDR specifically within compacted chromatin during the G2 phase of the cell cycle [98]. In contrast, the SIRT6/SNF2H complex is indispensable for NHEJ-mediated repair during the G1 phase NHEJ [98]. Collectively, these findings establish SIRT6 as a central regulator of chromatin accessibility and a promoter of efficient DSB repair [Fig. 3].

### Glucose metabolism

**Fig. 4:**
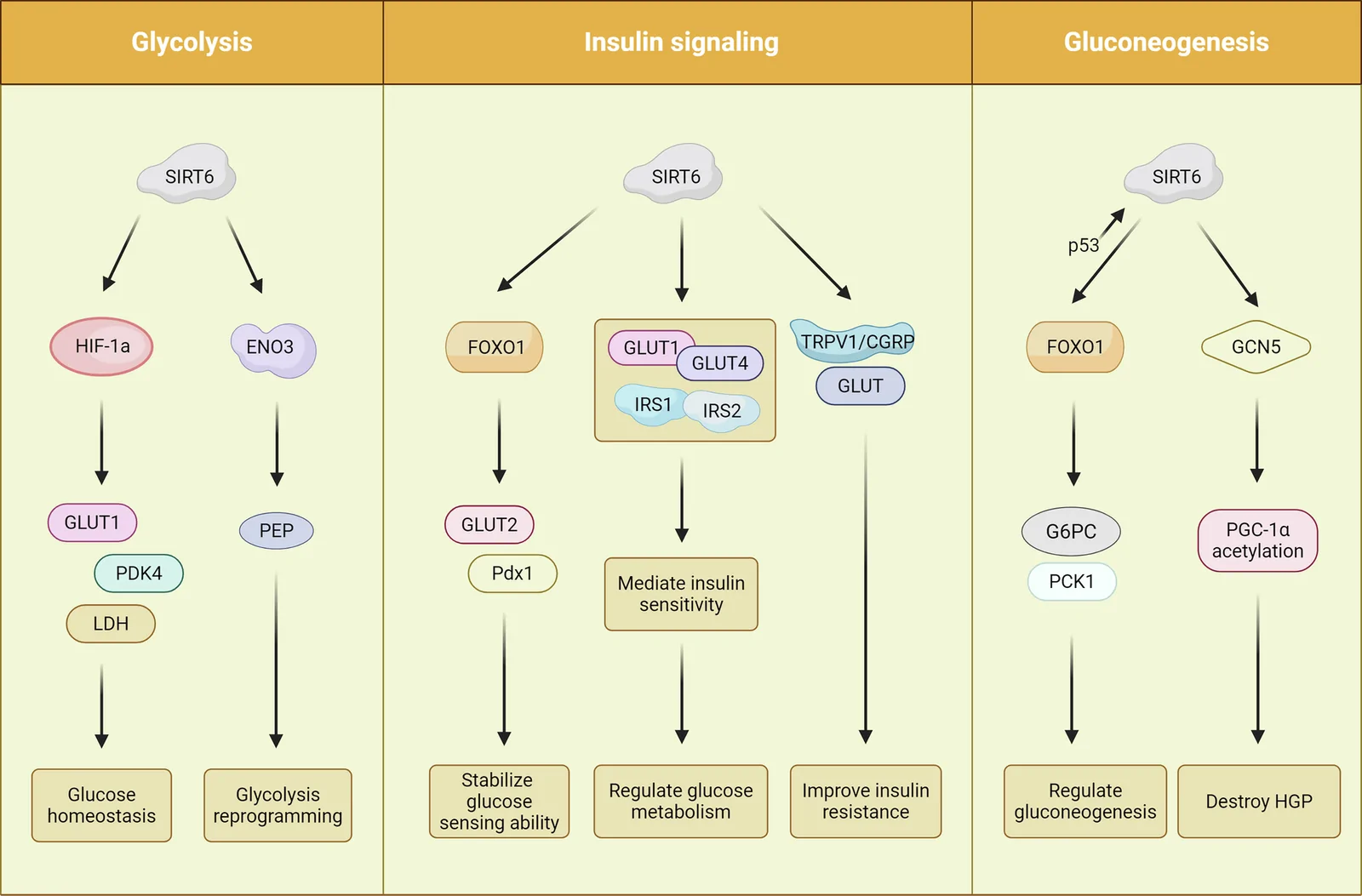
Key functions of SIRT6 in glucose metabolism regulation. HIF1α hypoxia-inducible factor 1, GLUT1 glucose transporter 1, PDK4 pyruvate dehydrogenase kinase 4, LDH lactate dehydrogenase, ENO3 enolase 3, PEP phosphoenolpyruvic acid, FOXO1 forkhead box protein O1, GLUT2 glucose transporter 2, Pdx1 pancreatic duodenal homeobox 1, IRS1/IRS2 Insulin Receptor Substrate 1 and 2, TRPV1 transient receptor potential vallinoid 1, CGRP calcitonin gene-related peptide, PCK1 phosphoenolpyruvate carboxykinase, G6PC glucose-6-phosphatase, GCN5 general control nonrepressed protein 5, PGC-1α peroxisome proliferator-activated receptor γ coactivator 1-α.

The role of SIRT6 in maintaining glucose homeostasis was first demonstrated in SIRT6-deficient mice, which exhibited severe metabolic disorders at an early stage, including fatal hypoglycemia [87]. Subsequent studies revealed that SIRT6 regulates glucose metabolism through complex mechanisms, including glycolysis, insulin signaling, and gluconeogenesis, thereby maintaining glucose homeostasis (Fig. 4).

In terms of glycolysis, Zhong et al. confirmed that SIRT6 regulates the expression of multiple glycolysis-related genes by catalyzing the deacetylation modification of histone H3K9 [53]. Specifically, SIRT6 inhibits the transcription factor HIF-1α, a core regulator of nutrient stress responses, and SIRT6-deficient cells exhibit upregulated HIF-1α expression and glycolysis. SIRT6 inhibits the expression of numerous key glycolytic genes by deacetylating histone H3K9 in their promoter regions. These genes include glucose transporter 1 (GLUT1), pyruvate dehydrogenase kinase 4, and lactate dehydrogenase. Moreover, cytoplasmic SIRT6 accumulation promotes its interaction with its downstream target enolase 3 (ENO3), directly driving ENO3 deacetylation and significantly enhancing the production of phosphoenolpyruvic acid, which in turn triggers glycolytic reprogramming [99].

SIRT6 also regulates the expression of key molecules in insulin signaling. Mice with physiological SIRT6 overexpression are protected against hyperglycemia and glucose intolerance induced by a high-calorie diet, suggesting that moderate SIRT6 overexpression enhances insulin sensitivity [100]. In pancreatic β-cells, SIRT6 knockdown results in a significant downregulation of glucose-stimulated insulin secretion [101]. Furthermore, SIRT6 stabilizes glucose-sensing ability in pancreatic β-cells by promoting FOXO1 deacetylation and the transcription of glucose-sensitive genes, such as GLUT2 and pancreatic duodenal homeobox [102]. In contrast, SIRT6 deficiency promotes membrane binding of the glucose transporters GLUT1 and GLUT4 and potentiates insulin signaling through insulin receptor substrates 1 and 2, resulting in hypoglycemia [103]. In model mice fed a high-fat diet, SIRT6 overexpression positively regulates the transient receptor potential vallinoid 1/calcitonin gene-related peptide signaling axis, upregulating the protein and mRNA expression of GLUT and ultimately enhancing glucose uptake and attenuating insulin resistance [104]. Tang et al. reported that SIRT6 decreases the stability of ERα protein through a P300-dependent mechanism, thus impairing estrogen-induced insulin signaling and sensitivity in the liver. These findings suggest that SIRT6 impacts insulin sensitivity in a sex-dependent manner [105].

SIRT6 is also implicated in the maintenance of glucose homeostasis by inhibiting gluconeogenesis. In a study involving multiple cancer cell lines, p53-induced activation of SIRT6 was revealed to directly interact with FOXO1, subsequently promoting its deacetylation and its translocation from the nucleus to the cytoplasm. This translocation effectively downregulates the expression levels of two rate-limiting enzymes in gluconeogenesis, including phosphoenolpyruvate carboxykinase (PCK1) and glucose-6-phosphatase (G6PC) [106]. Mechanistically, when FOXO1 is localized to chromatin, it stimulates gluconeogenesis by associating with the promoters of G6PC and PCK1, whereas SIRT6-mediated deacetylation initiates FOXO1 [106]. Moreover, SIRT6 interacts with GCN5 to modify and enhance its activity, promotes the acetylation of PGC-1α (a vital factor in gluconeogenic gene transcription), and disrupts HGP. These data suggest that SIRT6 activation may improve insulin-resistant diabetes [66]. Consistently, a recent study on diabetic kidney tissues also demonstrated that SIRT6 influences the nuclear translocation of FOXO1 to modulate glucose reabsorption and gluconeogenesis [107].

### Lipid metabolism

**Fig. 5:**
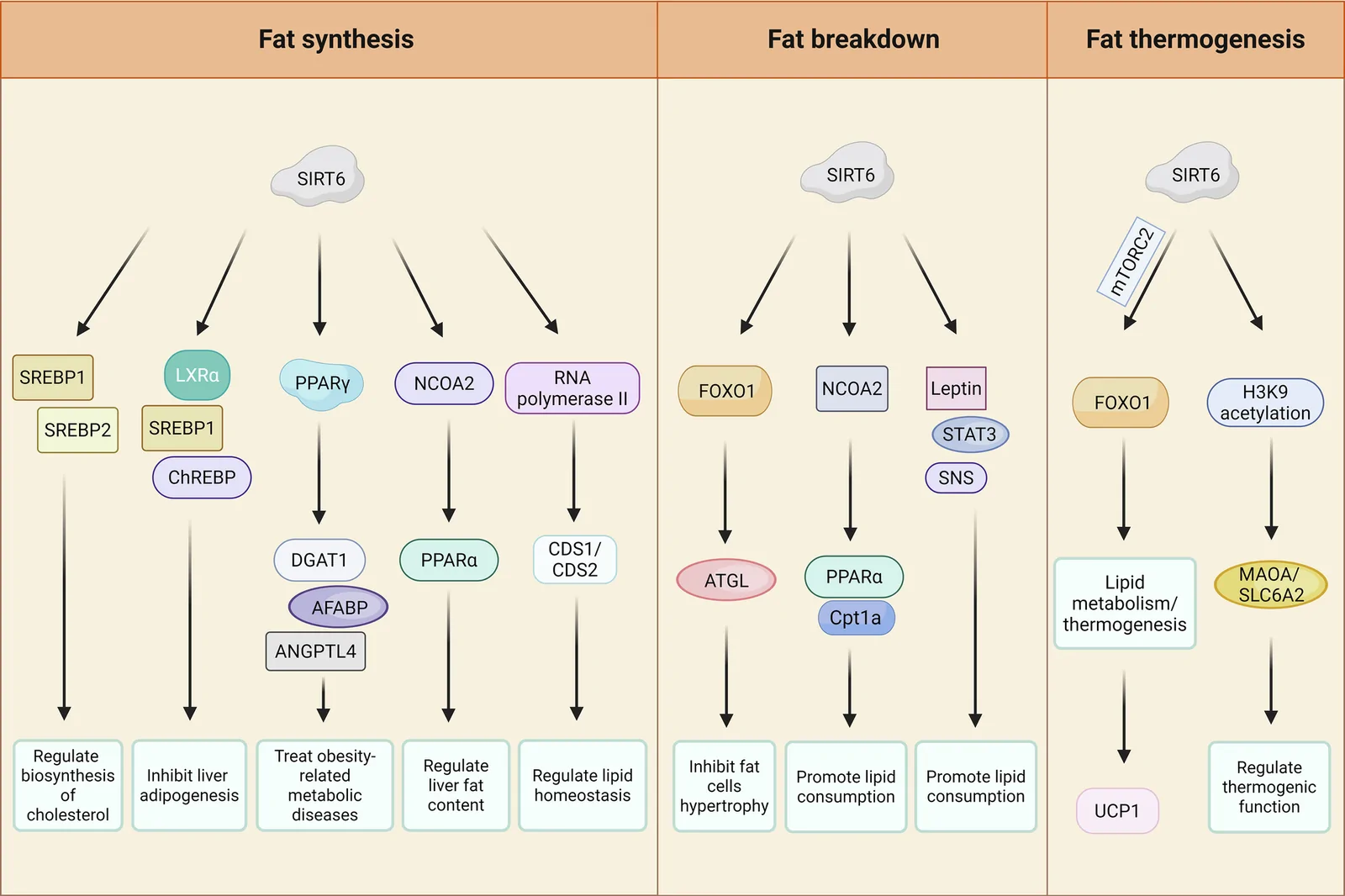
Key functions of SIRT6 in lipid metabolism regulation. SREBP1/SREBP2 sterol regulatory element-binding protein 1 and 2, LXRα liver X receptor α, ChREBP carbohydrate response element binding protein, PPARγ peroxisome proliferator-activated receptor γ, DGAT1 diacylglycerol acyltransferase 1, AFABP adipocyte fatty acid binding protein, ANGPTL4 angiopoietin-like protein 4, NCOA2 nuclear receptor coactivator 2, CDS1/CDS2 CDP-diacylglycerol synthase 1/2, ATGL adipose triglyceride lipase, Cpt1a carnitine palmitoyl transferase 1a, STAT3 signal transducer and activator of transcription 3, SNS sympathetic nervous system, UCP1 uncoupling protein 1, MAOA monoamine oxidase A, SLC6A2 solute carrier family 6 member 2.

Recently, SIRT6 has been identified as a key molecule in the regulation of lipid metabolism (Fig. 5). A mouse model with hepatic SIRT6 deficiency exhibited triglyceride accumulation, a fatty liver phenotype, and decreased β-oxidation [108]. However, SIRT6 overexpression significantly downregulated the synthesis of triglycerides and low-density lipoprotein cholesterol, reversing the upregulated expression of genes related to lipid storage following diet-induced obesity [109]. Through multiple mechanisms, SIRT6 contributes to the control of lipid synthesis, degradation, and transport, plays a crucial role in various metabolic diseases, and serves as a core factor in maintaining lipid homeostasis [110].

Sivan et al. reported that SIRT6 can negatively regulate SREBP1 and SREBP2 through multiple mechanisms, mainly the following: (a) directly inhibition of the transcription of sterol regulatory element-binding protein 1 and 2 (SREBP1/SREBP2) and their target genes; (b) prevention of the cleavage of the SREBP1/SREBP2 complex into its active form; and (c) upregulation of the SREBP1 phosphorylation and suppression of SREBP1 activity through AMPK activation [111]. These findings demonstrate that SIRT6 acts on lipogenic transcription factors to regulate cholesterol biosynthesis. Furthermore, FOXO3 recruits SIRT6, which deacetylates H3K9 and H3K56 in the SREBP2 promoter region and regulates its transcriptional activity [112]. Compared with WT mice, mice with hepatic SIRT6 knockout developed more severe fatty liver under the same dietary conditions [113]. Mechanically, SIRT6 inhibits the transcriptional activity of liver X receptor α, SREBP1, and carbohydrate response element-binding protein via deacetylation, conferring a protective effect against liver adipogenesis [113]. SIRT6 can also interact with PPARγ and downregulate genes related to lipid storage, such as diacylglycerol acyltransferase 1, adipocyte fatty acid binding protein, and angiopoietin-like protein, indicating that SIRT6 may be implicated in metabolic diseases [109]. Another study reported that SIRT6 inhibits SREBP-dependent triglyceride and cholesterol biosynthesis via PPARα, in which the PPARα coactivator NCOA2 is also involved [114]. In the context of cardiolipin synthesis, SIRT6 interacts with p53 and effectively recruits RNA polymerase II to the promoters of CDP-diacylglycerol synthase 1/2 (CDS1/CDS2) to maintain lipid homeostasis [115].

SIRT6, as a key factor in the fat synthesis process, also plays a crucial role in fat breakdown. In mice with fat-specific SIRT6 knockout, the activity of adipose triglyceride lipase (ATGL), a key enzyme in the fat breakdown process, is decreased, leading to fat cell hypertrophy [116]. Mechanistically, the enhanced acetylation and phosphorylation of FOXO1 interferes with the transcriptional level of ATGL, resulting in the inhibition of ATGL activity. Moreover, SIRT6 promotes the deacetylation of the liver PPARα coactivator NCOA2 K780 and induces the expression of PPARα and its target gene carnitine palmitoyl transferase 1, thus mediating hepatic lipid consumption and degradation [114]. In hypothalamic pro-opiomelanocortin (POMC) neurons, SIRT6 activates leptin-induced signal transducer and activator of transcription 3 (STAT3) and promotes leptin-dependent fat breakdown [117]. In addition, SIRT6 promotes the activity of the sympathetic nervous system, and this effect is closely linked to adipose tissue browning and lipolytic activity [117]. Notably, supra-physiological SIRT6 overexpression may reduce STAT3 acetylation and POMC expression, which further exacerbates diet-induced obesity and metabolic disorders [118]. These data suggest that balanced SIRT6 activity is crucial for maintaining metabolic homeostasis.

The thermogenic mechanism of brown adipose tissue significantly enhances energy expenditure, making it an interesting target for treating metabolism-related diseases. In fat tissues, SIRT6 overexpression can stimulate the thermogenic process, whereas its downregulation decreases the expression and activity of thermogenic genes, indicating that SIRT6 regulates fat thermogenesis [119]. The interaction between SIRT6 and mTOR complex 2 regulates FOXO1deacetylation, promotes lipid metabolism and thermogenesis, and upregulates UCP1 expression [120]. A recent study indicated that myeloid-specific SIRT6 deficiency facilitates H3K9 acetylation in the promoter region and the expression of genes encoding monoamine oxidase A and solute carrier family 6 member 2, resulting in decreased norepinephrine concentrations and impaired thermogenic function in brown adipose tissue [121].

## Sirt6 and kidney diseases

SIRT6 plays a crucial role in kidney homeostasis and disease progression through multiple interconnected mechanisms, such as lipid metabolic homeostasis, glucose metabolic homeostasis, inflammatory response, and cellular senescence, and is closely related to various kidney diseases (Table 2). Research on SIRT6 in kidney diseases has primarily focused on several cell types, including renal tubular epithelial cells (RTECs), podocytes, mesangial cells, and renal cancer cells (Table 3), collectively advancing our understanding of the role of SIRT6 in kidney disease progression. The following section systematically reviews the research progress across various kidney diseases.

**Table 2:** The role of SIRT6 in various kidney diseases.

Kidney diseases	In vitro	In vivo	Mechanism	Biologic effects	References
AKI	primary proximal tubule epithelia cells with 25 mM CP	cisplatin-induced AKI	catalyze the deacetylation of H3K9, suppress the expression level of ERK1/2	modulate inflammatory and apoptotic	[124]
AKI	HK-2 cells with 5 μM cisplatin	cisplatin-induced AKI	reduce the acetylation of histone H4K9, inhibit the transcription of BAP1	inhibit iron ptosis and alleviate kidney damage	[123]
AKI	HK-2 with 5, 10 and 50 μg/mL LPS for 48 h	AKI model with LPS using mice	reduce the expression of TNF-α and IL-6	inhibit apoptosis, promote autophagy, contribute to the repair process of LPS-induced renal injury	[125]
AKI	HK-2 with LPS (1 μg/mL)	AKI model with LPS using mice	inhibit the downstream ACMSD pathway	promote lipid metabolism and reduce apoptosis	[126]
AKI	LPS-induced HK-2 cells	sepsis mouse model established by CLP	USP10 trigger NRF2/ARE pathway by SIRT6	down-regulate apoptosis and oxidative stress, and alleviate renal function impairment	[127]
AKI	Hypoxia-induced TECs	Ischemia/reperfusion -induced AKI	regulate the arrest of the G2/M phase	avoid TECs damage triggered by hypoxia	[128]
AKI	mouse kidney mesangial MES13E cells	cadmium-induced kidney toxicity	polyUb-p62 target Sirt6 to autophagosomes	play roles in CD-induced cell death and renal injury	[129]
AKI	/	vancomycin induced renal injury	block the HIPK2/p53 signaling	reduce the number of TUNEL-positive cells and increase SOD and GSH	[130]
DKD	podocytes with HG	STZ-induced type 1 diabetic mouse model	activate AMPK signaling pathway	reduce podocyte apoptosis and oxidative stress, and improve mitochondrial defects	[135]
DKD	human podocytes	STZ-induced DKD in mice	suppress the transcription of Notch1 and Notch4 by catalyzing the deacetylation of H3K9	maintain the podocyte cytoskeleton, decrease the expression of the urokinase plasminogen activator receptor, and improve proteinuria	[54]
DKD	human normal glomerular podocyte cell line with 30 mmol/l HG	/	regulate the Notch1 signaling pathway	protect against podocyte autophagy injury	[136]
DKD	podocytes with 50 mmol/L HG treatment	STZ-induced DKD rats	polarize macrophages into the M2 phenotype	inhibit apoptosis, and protect podocytes from HG-induced injury	[137]
DKD	human podocytes with 30 mM glucose	db/db mice	target the Nrf2/GPX4 signaling pathway	alleviate oxidative stress, improve mitochondrial dysfunction, and reduce iron ptosis and podocyte damage	[138]
DKD	human podocytes with 30 mM HG for 24 h	db/db mice	activate the PI3K/AKT signaling pathway	protect podocytes against HG-induced cytoskeletal remodeling and apoptosis	[139]
DKD	HK-2 cells	STZ-induced DKD mice	target NAMPT-SIRT6 axis	play an important role in the process of DKD fibrosis	[141]
DKD	HG-induced HK2 cells	db/db mice	Sirt6/Smad3 signaling pathways	protect against kidney injury in DKD	[142]
DKD	HG-induced TECs	STZ-induced DKD rats	repress Nlrp3 transcription by deacetylating H3K9	prevent TECs damage	[15]
DKD	HG-induced rat mesangial cells	STZ-induced DKD rats	modulate the miR-33a-5p/SIRT6 axis	alleviate the inflammatory response and fibrosis degree of the kidneys	[143]
Renal fibrosis	mouse proximal tubule (BUMPT) cells treated with TGF-β	UUO	modulate β-catenin signaling by deacetylating H3K56 at the promoters of β-catenin target genes	inhibit the transcription of genes linked to fibrosis	[148]
Renal fibrosis	HK2 cells treated with TGF-β1	mice with a high adenine diet	inhibit HIPK2 activity	reduce collagen deposit, collagen I and α-smooth muscle actin expression	[16]
Renal fibrosis	HK2 cells treated with TGF-β1 for 24 h	UUO	regulate β-catenin acetylation and ECM protein promoter transcription	play a role in tubulointerstitial inflammation and fibrosis	[149]
Renal fibrosis	mouse renal tubular epithelial cell line BUMPT treated with 10 ng/ml TGF-β	UUO	phosphorylation of SIRT6 to prevent its proteasomal degradation	alleviate renal fibrosis	[150]
HN	Ang II-stimulated podocytes	Ang II-treated rat	activate Nrf2/HO-1 pathway	alleviate Ang II-induced podocyte apoptosis and DSBs	[153]
HN	Ang II-cultured podocytes	Ang II-treated mice	mediate the ROCK1-Drp1 signaling pathway	protect against Ang II-induced mitochondrial fission and apoptosis in podocytes	[154]
HN	Ang II-cultured podocytes for 24 h	Ang II-treated mice	regulate PLD6 signaling	maintain podocyte CL metabolism and suppress Ang II-induced lipid accumulation	[156]
HN	Ang II-cultured podocytes for 24 h	Ang II-treated mice	mediate the expression of ABCG1	affect cholesterol efflux in podocytes	[157]
RCC	Human renal cancer cell lines (786-O)	/	result in the cessation of G1/S phase	lead to a reduction in cell viability and an increase in chemotherapy sensitivity	[161]
RCC	ccRCC-derived 769-P and 786-O cells	BALB/c nude mice	promote Bax expression, reduce Bcl-2	suppress cancer cell proliferation and invasion	[162]
Calcium oxalate nephrocalcinosis	HK − 2 with CaOx (100 μg/mL)	mice with glyoxylate injections of 100 mg/kg	activate NRF2	protect mitochondrial function, alleviate oxidative stress, and improve inflammatory response	[164]
Calcium oxalate nephrocalcinosis	HK-2 with CaOx monohydrate	mice with glyoxylic acid injection	down-regulate the TRL4/NF-κB signaling pathway	down-regulate the expression of IL-1β, TNF-α, MCP-1, and IL-6	[165]
Kidney senescence	WI-38 cells	the aged mice	/	reverse renal function	[168]
Kidney senescence	Primary murine podocytes	the aged mice	/	maintain glomerular function and podocyte homeostasis	[169]
MN	zymosan activation serum-treated podocytes	cationic bovine serum albumin- treated rats	mediate the Wnt1/β-catenin pathway	attenuate podocyte damage	[170]

**Table 3:** Cell-type-specific regulatory mechanisms of SIRT6 in kidney diseases.

SIRT6	**Cell types**	**Mechanisms**
	Renal tubular epithelial cells	Inflammation, apoptosis, ferroptosis, autophagy, lipid metabolism, oxidative stress, fibrosis, mitochondrial function
	Podocytes	Apoptosis, oxidative stress, mitochondrial function, autophagy, ferroptosis, actin cytoskeleton, lipid metabolism
	Mesangial cells	Autophagy, fibrosis, inflammation
	Renal clear cell carcinoma cells	G1/S phase arrest, apoptosis

### SIRT6 in AKI

The prevalence of AKI is rising rapidly, with an extremely high mortality rate. This burden has rendered it a global public health challenge. Moreover, AKI is a well-recognized contributor to the onset and progression of CKD [122]. Notably, SIRT6 downregulation is a consistent phenomenon observed in various AKI models, including those induced by cisplatin, sepsis, cadmium, and vancomycin. To date, research in this area has mainly focused on RTECs and mesangial cells, in which SIRT6 downregulation exacerbates kidney injury via multiple mechanisms. SIRT6 downregulation is an important driver of the onset of AKI, whereas its exogenous upregulation, such as through pharmacological activators or gene overexpression, significantly alleviates kidney injury [123]. Therefore, a deeper understanding of the intricate protective mechanism of SIRT6 in AKI may offer a theoretical foundation for targeted therapeutic strategies.

Overexpression of SIRT6 alleviates cisplatin-induced kidney injury by modulating inflammatory and apoptotic pathways both in vitro and in vivo [124]. Specifically, SIRT6 binds to the promoters of extracellular signal-regulated kinase1/2 (ERK1/2) and deacetylates H3K9 to downregulate the expression level of ERK1/2, thereby preventing the exacerbation of renal injury induced by SIRT6 knockout. In the cisplatin-induced AKI model, SIRT6 expression is significantly downregulated both at the mRNA and protein levels [124]. Cisplatin treatment promotes acetylation of histone H4K9 at the BAP1 promoter and alters the chromatin structure of BAP1 to promote its transcription. BAP1, in turn, triggers ferroptosis and aggravates renal injury [123]. Moreover, the use of UBCS039, a SIRT6 agonist, significantly attenuates cisplatin-induced cellular damage in vitro.

In a lipopolysaccharide (LPS)-induced AKI model, SIRT6 overexpression results in the downregulation of the expression levels of TNF-α and IL-6, inhibits apoptosis, and promotes autophagy, thereby contributing to the repair process of LPS-induced renal injury [125]. Similarly, in HK-2 cells, silencing SIRT6 drives apoptosis with concomitant reduction of autophagy. In addition, SIRT6 significantly promotes lipid metabolism and inhibits apoptosis in AKI by inhibiting the downstream amino–carboxylic semialdehyde dehydrogenase (ACMSD) pathway [126]. In LPS-stimulated HK-2 cells, ACMSD upregulation enhances cellular apoptosis and lipid droplet accumulation; notably, these effects can be effectively reversed by treatment with UBCS039. A sepsis mouse model established through cecal ligation and perforation exhibits decreased expression of the ubiquitin-specific peptidase 10 (USP10) in renal tissue [127]. In these septic mice, USP10 triggers the NRF2/ARE pathway through SIRT6 and inhibits apoptosis and oxidative stress in RTECS, thereby alleviating renal function impairment.

Hypoxia-challenged tubular epithelial cells (TECs) and the mouse renal ischemia/reperfusion injury model exhibit marked downregulation of SIRT6, which exacerbates G2/M phase arrest and hypoxia-induced cellular damage [128]. SIRT6 can protect TECs from hypoxia-induced damage by regulating G2/M phase cell cycle arrest, suggesting its potential as a therapeutic target for alleviating renal injury. A study on cadmium-induced kidney toxicity demonstrated that polyubiquitinated-p62 is involved in cell death and kidney injury by targeting Sirt6 for autophagic degradation and thus regulating cell apoptosis [129]. In vancomycin-induced renal injury, the expression levels of HIPK2 and p-p53 are significantly upregulated, whereas those of the antioxidant enzymes superoxide dismutase and glutathione are downregulated, accompanied by an increased number of TUNEL-positive cells [130]. Notably, SIRT6 overexpression considerably ameliorates these pathological changes and improves renal dysfunction by blocking HIPK2/p53 signaling.

### SIRT6 in chronic kidney diseases

#### SIRT6 in DKD

DKD is among the most prevalent and serious chronic microvascular complications of diabetes mellitus, mainly driven by long-term hyperglycemia and progressive damage to renal structure and function. It ultimately advances to end-stage renal disease (ESRD) and poses a substantial burden on the quality of life and health of patients [131–133]. Mechanistically, diabetes-associated metabolic changes inflict kidney damage through multiple mechanisms, manifesting histologically as glomerular hypertrophy, glomerular sclerosis, and renal tubular fibrosis [134]. Evidence from multiple DKD models established in recent years consistently demonstrates that SIRT6 influences the pathological progression of DKD via several distinct pathways [110], with relevant studies mostly focusing on podocytes, RTECs, and mesangial cells. Collectively, the ability of SIRT6 to regulate cellular senescence and glycolipid metabolic homeostasis holds considerable value in the prevention and treatment of DKD.

In podocytes overexpressing SIRT6, high-glucose (HG)-induced oxidative stress and podocyte apoptosis are significantly attenuated, and HG-induced mitochondrial dysfunction is effectively ameliorated, mainly through AMPK signaling pathway activation [135]. SIRT6 also suppressed HG-induced increases in H3K9ac and H3K56ac levels. Fan et al. discovered that SIRT6 protects podocytes from injury in murine models of DKD and adriamycin-induced nephropathy through epigenetic regulation of Notch signaling [54]. Mechanistically, SIRT6 suppresses Notch1 and Notch4 transcription via H3K9 deacetylation. This suppression leads to the preservation of the podocyte cytoskeleton and decreased expression of the urokinase plasminogen activator receptor, thereby alleviating proteinuria. In addition, SIRT6 mitigates podocyte injury in DKD by regulating Notch1-mediated autophagy [136]. SIRT6 can induce the polarization of macrophages toward the M2 phenotype, inhibit apoptosis, and protect podocytes from HG-induced injury, suggesting its role as an immunoregulatory factor in DKD pathogenesis [137]. In HG-treated human podocytes, SIRT6 effectively attenuates oxidative stress responses, restores mitochondrial dysfunction, and alleviates ferroptosis and podocyte damage by targeting the Nrf2/GPX4 signaling pathway [138]. In addition, SIRT6 acts on the PI3K/AKT pathway to protect podocytes from HG-induced cytoskeleton remodeling and apoptosis [139].

Nicotinamide mononucleotide (NMN), a precursor of NAD⁺, can induce the onset of diabetic proteinuria when its secretion level in the proximal tubule decreases [140]. Mice with proximal tubule-specific knockout of nicotinamide phosphoribosyltransferase (Nampt), which is produced by NMN, demonstrate collagen deposition and downregulated expression of sirtuins, particularly SIRT6. These findings suggest that the NAMPT-SIRT6 axis in the proximal tubule plays an important role in the progression of DKD fibrosis [141]. Moreover, SIRT6 downregulation in the kidneys of db/db mice results in progressive epithelial-mesenchymal transition and tubular dysfunction. Mechanistically, FOXO3a transactivates SIRT6 expression by binding to its promoter; subsequently, SIRT6 directly binds and deacetylates SMAD3, a key downstream mediator of TGF-β signaling, thereby inhibiting the transcriptional activity of SMAD3 in HG-induced HK2 cells [142]. SIRT6 also directly represses NLRP3 transcription through H3K9 deacetylation, which in turn inhibits NLRP3 inflammasome activation and tubular injury, confirming SIRT6 as a protective epigenetic mediator in DKD [15]. At the post-transcriptional regulatory level, Jiang et al. reported that circular RNA ITCH alleviates the inflammatory response and fibrosis in the kidneys of diabetic mice by modulating the miR-33a-5p/SIRT6 axis [143].

#### SIRT6 in renal fibrosis

Renal fibrosis is a common feature of almost all CKDs and a hallmark presentation of progression to ESRD [144]. The typical morphological features of renal fibrosis include structural remodeling and aberrant deposition of renal tissue, such as thickening of the glomerular basement membrane, expansion of the mesangial matrix, glomerular sclerosis, RTEC atrophy, and renal interstitial fibrosis [145]. Severe injury to the renal tubules may trigger aberrant repair, accelerate the progression from AKI to CKD, and promote the progression of renal fibrosis [146]. Importantly, renal fibrosis is not a static process but a dynamically evolving pathological condition involving a series of complex cellular events, such as the inhibition of matrix degradation, activation of fibroblasts, and infiltration of immune cells [147]. Several studies have shown that SIRT6 is involved in the progression of renal fibrosis. Therefore, the possibility of SIRT6 delaying the progression of CKD by inhibiting renal fibrosis deserves in-depth exploration.

In the fibrotic process following obstructive nephropathy and kidney ischemia-reperfusion injury, SIRT6 expression is significantly upregulated. This pattern is similarly observed in mouse proximal tubule cells treated with TGF-β. During TGF-β stimulation, SIRT6 interacts with β-catenin, binding to the promoter of β-catenin to drive H3K56 deacetylation, thereby inhibiting the transcription of fibrosis-related genes [148]. Compared with WT mice fed an adenine-rich diet, Sirt6-transgenic mice exhibit significantly decreased collagen deposition and reduced expression of α-smooth muscle actin and type I collagen under identical dietary conditions. The underlying mechanism may involve SIRT6-mediated inhibition of HIPK2 activity to alleviate renal interstitial fibrosis [16]. Proximal tubular SIRT6 is also involved in UUO-induced tubulointerstitial inflammation and fibrosis by modulating β-catenin deacetylation and TGF-β1/Smad signaling and reducing extracellular matrix production [149]. In addition, GSK-3β, a phosphorylated kinase, interacts with SIRT6 to prevent its proteasomal degradation, thereby alleviating renal fibrosis [150]. Notably, current research on the regulatory mechanisms of SIRT6 in renal fibrosis is mainly based on RTECs, warranting future investigations employing a wider range of renal cell types to systematically elucidate the regulatory network of SIRT6 in renal fibrosis.

#### SIRT6 in HN

Long-term hypertension is a well-established risk factor for structural and functional renal damage [151]. Specifically, persistent hypertension can cause continuous injury to the glomeruli, renal tubules, and vasculature within the kidneys, triggering a cascade of pathological reactions encompassing renal cell apoptosis, scar tissue formation, and impaired filtration function [152]. Therefore, HN is an important driving force underlying the onset of CKD and ESRD. SIRT6 injury in HN progression by regulating key pathological processes, such as mitochondrial function, lipid metabolism, and apoptosis.

In the glomeruli of patients with HN, downregulated SIRT6 expression is accompanied by a concurrent rise in DSBs. Similar findings are also observed in Ang II-treated rat kidney tissues and podocytes [153]. Furthermore, SIRT6 alleviates Ang II-induced podocyte apoptosis and DSB accumulation by transcriptionally activating NRF2 and HO-1 [153]. In Ang II-induced in vivo and in vitro models, Chen et al. reported that specific SIRT6 knockout significantly exacerbates podocyte apoptosis and the mitochondrial fragmentation phenomenon. Mechanistically, these effects are closely related to the SIRT6-mediated Rho-associated coiled coil-containing protein kinase 1/dynamin-related protein 1 signaling [154]. Moreover, aberrant cardiolipin metabolism is directly or indirectly involved in renal pathological injury [155]. In particular, SIRT6 may maintain podocyte cardiolipin metabolism and suppress Ang II-induced lipid accumulation through phospholipase D family member 6 (PLD6) signaling, suggesting the potential of the SIRT6–PLD6 axis as a therapeutic target for lipid nephrotoxicity [156]. In another study, podocyte-selective SIRT6 deletion was demonstrated to significantly promote cholesterol accumulation by mediating ATP-binding cassette transporter G1, ultimately exacerbating Ang II-induced renal injury [157].

### SIRT6 in other renal diseases

In the past decade, beyond its role in AKI, DKD, renal fibrosis, and HN, research on SIRT6 has comprehensively explored its role in RCC, kidney senescence, and calcium oxalate nephropathy.

RCC occurs in RTECs and is a common malignant tumor in the urinary system, accounting for approximately 90% of renal malignant tumors [158]. In a study involving 102 samples obtained during renal mass resection surgeries, comparisons of the expression differences of the sirtuin family in RCC and adjacent normal renal tissues revealed significant downregulation of SIRT1, SIRT3, and SIRT6 expression in RCC tissues [159]. However, this trend is not consistent across all studies. For example, another study indicated that SIRT6 is highly expressed in clear cell RCC (ccRCC) [160]. In RCC, SIRT6 deficiency promotes G1/S phase arrest, reduces cell viability, and enhances chemotherapy sensitivity [161]. In 769-P and 786-O cells, SIRT6 deletion promotes Bax expression, decreases B-cell lymphoma 2 expression, and suppresses cancer cell proliferation and invasion, indicating that SIRT6 functions as a proto-oncogene in ccRCC [162]. Therefore, in contrast to its protective role in non-malignant kidney diseases, SIRT6 may exert opposite effects in RCC.

Aberrant deposition of calcium oxalate crystals resulting from metabolic dysregulation within the renal parenchyma causes damage to renal structure and function [163]. Overexpression of SIRT6 can preserve mitochondrial function and alleviate oxidative stress damage, significantly improving the infiltration of inflammatory cells both in vivo and in vitro [164]. Mechanistically, SIRT6 mitigates the inflammatory response and oxidative stress caused by calcium oxalate nephrocalcinosis by activating NRF2, providing a mechanistic basis for the prevention and treatment of this condition [164]. MDL-800, a selective SIRT6 allosteric agonist, can inhibit the infiltration of inflammatory cells in mice with calcium oxalate nephrocalcinosis by enhancing the deacetylase activity of SIRT6 and downregulating the Toll-like receptor 4 (TLR4)/NF-κB signaling pathway [165].

Cellular senescence is an irreversible state that can involve all types of kidney cells [166]. Accumulating evidence has confirmed that SIRT6 delays cellular senescence by modulating oxidative stress and preserving telomere integrity [167]. In aged mice, SIRT6 silencing induces NF-κB signaling and contributes to cellular senescence [168]. However, caloric restriction markedly restores renal function and upregulates SIRT6 expression [168]. Moreover, SIRT6 deficiency in aging mice results in kidney inflammation and some pathological changes, including renal hypertrophy and glomerular enlargement, suggesting the significance of SIRT6 in age-related renal dysfunction [169].

In a recent study, renal SIRT6 expression was observed to be significantly downregulated in patients with membranous nephropathy, and this finding was further confirmed in cationic bovine serum albumin-stimulated rats and zymosan activation serum-treated podocytes [170]. A subsequent study revealed that SIRT6 attenuates podocyte damage by modulating the Wnt1/β-catenin pathway.

## Sirt6 modulators

Given that SIRT6 is involved in the biological processes of various kidney diseases, regulating SIRT6 activity via small modulators is expected to emerge as a potential strategy for the prevention and treatment of kidney diseases. The SIRT6 modulators can be classified into direct and indirect modulators based on their mode of action: direct modulators directly bind SIRT6 and regulate its enzymatic activity, whereas indirect modulators indirectly regulate SIRT6 expression or function by affecting upstream signaling pathways or transcriptional levels. A detailed overview of SIRT6 modulators associated with kidney diseases is presented in Table 4.

**Table 4:** SIRT6 as modulators in kidney diseases.

Classification	Modulators	Structure	Cell	Disease	Pathway	Biologic effects	References
Natural products	Isoorientin		mTECs	AKI	SIRT6/Nrf2	attenuate inflammatory responses, oxidative stress, and apoptosis processes	[172]
	Daphnetin		HK-2	AKI	SIRT6/Nrf2	inhibit NOX4, the apoptotic pathway, and the NF-κB and MAPK inflammatory pathways	[173]
	Polydatin		HK-2	AKI	/	target restoration of SIRT6-regulated autophagic flux	[174]
	Astragaloside IV		podocyte	DKD	SIRT6/HIF-1α	ameliorate high glucose-induced podocyte pyroptosis	[175]
	Diosgenin		MPC5	DKD	PDK4/ANGPTL4	protect against podocyte injury in early phase of DN	[176]
	Ginsenoside Rb3		CIHP-1	obesity-mediated renal injury	PPARδ/SIRT6	attenuate inflammatory responses, oxidative stress, and apoptosis	[177]
	Oleuropein		/	sunitinib-induced nephrotoxicity	SIRT6/Notch-1/NLRP3/IL-1β	improve renal histology	[178]
Chemical synthesized compounds	MDL800		HK-2	calcium oxalate nephrocalcinosis	TRL4/NF-κB/ SIRT6	reduce the inflammatory and renal injury	[165]
	UBCS039		HK-2	AKI	SIRT6/ ACMSD	promote lipid metabolism and inhibit apoptosis	[126]
	OSS_128167		podocyte	DKD	/	aggravate podocyte damage	[139]
NAD^+^ promoter	Nampt		HK-2	DKD	Nampt-Sirt6	exhibit TBM thickening and collagen deposition	[141]

In the field of drug development, natural products serve as extremely valuable sources of compounds, owing to their diverse structures and wide range of biological activities [171]. Natural products play a key role in kidney protection by indirectly modulating SIRT6 activity, effectively preventing the progression of various kidney diseases. For example, isoorientin can activate the Nrf2 pathway mediated by SIRT1 and SIRT6 and attenuate inflammatory responses, oxidative stress, and apoptosis processes, thus exerting a significant renal protective effect in cisplatin-induced AKI [172]. Furthermore, daphnetin alleviates cisplatin-induced nephrotoxicity by inhibiting NOX4 expression, the apoptotic pathway, and the NF-κB and MAPK inflammatory pathways, while upregulating SIRT6 expression [173]. Polydatin, a polyphenolic compound, effectively delays the progression of AKI through the targeted restoration of SIRT6-regulated autophagic flux [174]. Astragaloside IV, an active ingredient with established renal protective effects, significantly attenuates HG-induced podocyte pyroptosis by modulating the SIRT6/HIF-1α signaling pathway, thereby relieving renal injury in DKD [175]. Diosgenin protects against podocyte damage in DKD by regulating the function of SIRT6, as demonstrated in both in vitro and in vivo experiments [176]. In palmitate-treated human podocytes, Ginsenoside Rb3 significantly attenuates inflammatory responses, oxidative stress, and apoptosis by relying on PPARδ and SIRT6 signaling, suggesting its potential in delaying obesity-mediated renal injury [177]. Oleuropein, a major polyphenolic component in olive trees, exerts a protective effect against sunitinib-induced nephrotoxicity by engaging the SIRT6/Notch-1/NLRP3/IL-1β axis [178].

Several synthetic small molecules have been identified as direct modulators that specifically modulate SIRT6 enzymatic activity. Among these, MDL800, an SIRT6 selective agonist, is involved in numerous biological processes, such as cell senescence and DNA damage repair. In mice with calcium oxalate nephrocalcinosis, MDL-800 can drive histone deacetylation and inhibit the TLR4/NF-κB signaling pathway, significantly mitigating the inflammatory response and renal injury [165]. UBCS039, a selective small-molecule agonist of SIRT6, activates the deacetylase activity of SIRT6 by specifically binding to its acyl channel pocket. In AKI, UBCS039 can promote lipid metabolism and inhibit apoptosis by targeting the downstream molecule ACMSD [126]. OSS_128167, also known as SIRT6-IN-1, is a selective small-molecule inhibitor of SIRT6 that is widely used in studies investigating the functions of SIRT6. Notably, OSS_128167 aggravates podocyte damage under HG conditions [139].

As previously described, the activity of SIRT6 depends on NAD^+^ and NMN. NAMPT, a key rate-limiting enzyme, can determine the efficiency of NAD^+^ generation. Proximal tubule-specific Nampt conditional knockout mice exhibit SIRT6 downregulation, collagen deposition, and tubular basement membrane thickening; these pathological changes are attributed to the upregulation of TIMP-1 [141].

SIRT6 modulators hold significant research value and translational potential for the prevention and treatment of kidney diseases. However, based on current evidence, substantial challenges remain. At present, natural compounds, such as isoorientin, daphnetin, astragaloside IV, and ginsenoside Rb3, remain confined to the preclinical stage. Although diosgenin, oleuropein, and polydatin have advanced into human clinical studies, their investigation has not yet extended to kidney diseases [179–181]. Similarly, small-molecule modulators such as MDL-800, UBCS039, and OSS_128167 also lack supporting clinical trial evidence. Therefore, future efforts should prioritize systematic research on SIRT6 modulators, focusing on elucidating their modes of action, levels of evidence, and disease-specific applicability. Such studies could promote the clinical translation of SIRT6 modulators and provide novel therapeutic strategies for kidney diseases.

## Conclusion and future perspective

As a critical histone deacetylase, SIRT6 serves as a vital guardian of cellular homeostasis by maintaining genomic stability and protecting against endogenous and exogenous stress, while simultaneously acting as a central regulator of renal homeostasis. Accumulating evidence indicates that SIRT6 deficiency triggers a cascade of cellular dysregulation, culminating in aberrant cell functions and thereby the progression of various kidney diseases. SIRT6 expression is significantly dysregulated across a spectrum of kidney disorders, including AKI, CKD, HN, and RCC. Notably, the functional importance of SIRT6 activity has been consistently demonstrated across various animal and cellular models, emphasizing its potential value in kidney diseases. Deepening mechanistic investigation of SIRT6 has further revealed its complex roles. For example, in the tumor microenvironment of RCC, SIRT6 may paradoxically promote the proliferation of cancer cells. In contrast, it exerts predominantly protective effects and ameliorates the disease status in AKI and CKD. Furthermore, as current research on SIRT6 in renal diseases has mainly focused on RTECs, podocytes, and mesangial cells, our systematic understanding of the specific regulatory network mediated by SIRT6 in different cell types, such as glomerular endothelial cells and macrophages, remains limited. Future studies should employ single-cell sequencing and spatial transcriptomics to systematically analyze the differential functions of SIRT6 across various renal cell types and further explore the intercellular crosstalk mechanisms among different types of renal cells. Such research could help establish a comprehensive regulatory map of SIRT6 within the pathological microenvironment. In parallel, acetylome-based proteomics should be utilized to screen key deacetylating proteins regulated by SIRT6, followed by direct target validation using techniques such as co-immunoprecipitation, in vitro deacetylation experiments, and site mutation to clarify the direct regulatory role of SIRT6 in specific renal cell types.

Over the past decade, substantial progress has been made in elucidating the biological functions of SIRT6. Nevertheless, the development of safe and effective modulators of SITR6 remains at the preliminary stage. Notably, the SIRT6 agonist (SP-624) has advanced from preclinical research to clinical trials, establishing a critical precedent for the development of small molecules that selectively target SIRT6 [182]. While SIRT6 modulators effectively improve key metrics in vivo models and in vitro models, conclusive clinical evidence of their efficacy in delaying the occurrence and progression of kidney disease in humans remains lacking. To address clinical translation challenges associated with SIRT6 regulators, future research should focus on achieving breakthroughs through multidimensional optimization. For example, previously verified regulators should be prioritized as potential lead compounds whose selectivity and pharmacokinetic properties can be optimized. Moreover, renal cell-targeted delivery systems, particularly those based on nanoparticles, should be further developed to effectively minimize off-target effects and systemic toxicity. Finally, designing high-quality clinical trials to clarify the efficacy and safety of these drugs is essential to comprehensively evaluate their stability, provide a basis for rational drug use, and ultimately realize the clinical potential of SIRT6-targeted strategies in the treatment of kidney diseases.
